# Pharmacists’ perception about efficacy, safety, and quality of dietary supplements that used for rheumatic disorders in the Iraqi pharmaceutical market

**DOI:** 10.1371/journal.pone.0306380

**Published:** 2024-07-25

**Authors:** Ali Haidar Al-Hadi, Ehab Mudher Mikhael

**Affiliations:** 1 Fourth Stage Student, College of Pharmacy, University of Baghdad, Baghdad, Iraq; 2 Clinical Pharmacy Department, College of Pharmacy, University of Baghdad, Baghdad, Iraq; Lahore Medical and Dental College, PAKISTAN

## Abstract

Rheumatic diseases are common progressive diseases that associated with chronic pain. Most patients seek to reduce the pain of these diseases by using dietary supplements (DS). Yet, most DS have limited benefits to reduce pain and/or disease progression. Therefore, this study aimed to explore the perceptions of community pharmacists about the efficacy, safety, and quality of the DS for treatment of rheumatic diseases (DSTRD) in the Iraqi pharmaceutical market. A qualitative study was conducted during February-2023 through face-to-face interview with community pharmacists with ≥6 months of working experience. The interviews were guided through semi-structured open-ended questions. The interviews were recorded using iPhone-11. A hybrid framework-model was used to analyze the data. Interviews were performed with 20 pharmacists. Only 30% of pharmacists considered DSTRD to be effective, whereas 75% of them perceived DSTRD to be safe. One-quarter of the participants considered the quality of DSTRD to be good. All interviewed pharmacists reported that prescribing DSTRD become a common practice. Eleven participants reported that deals with pharmaceutical companies are the main trigger for prescribing DSTRD. Three-quarter of participating pharmacists mentioned that they educate their patient about the dispensed DSTRD. However, only 10% of them educate patients about the possible side effects and interactions of DSTRD. In conclusion, most of the participating pharmacists have doubts about the efficacy, safety, and quality of DSTRD in the Iraqi market. The prescribing of such products by physicians is common and usually triggered by deals with pharmaceutical companies. Pharmacist-education to the patient on these supplements is poor.

## Introduction

Rheumatic disorders (RD) are common complaints for many patients seeking medical advice. The main RD include osteoarthritis (OA), rheumatoid arthritis (RA), gout, psoriatic arthritis, systemic lupus erthymatosus, ankylosing spondylitis, polymylagia rheumatica, giant cell arteritis, and sjögren’s syndrome. Meanwhile, OA and RA are the most common of these disorders in clinical practice [1, 2]. Both OA and RA are characterized by their chronic nature [3, 4] and usually associated with pain, functional limitation, and even disabilities which ultimately reducing patient quality of life [5]. Furthermore, RA is a progressive diseases, thus disease modifying antirheumatic drugs (DMARDs) should be initiated immediately upon diagnosis to mitigate disease progression [6]. However, most of DMARDs are expensive, slow in their action, and associated with serious side effects [7]. On the other hand, OA is also a progressive disease that can progress despite the usage of traditional analgesic therapy [8]. Therefore, most patients seek to reduce the pain of these progressive diseases by alternative and complementary medicines especially dietary supplements (DS) [9–11]. Meanwhile, a lot of DS are available in pharmacies and can be purchased easily without prescription in Iraq and in many other developing countries [12]. In addition, the prescription of DS for many chronic diseases including rheumatological diseases is very common among Iraqi physicians [13]. Unfortunately, most DS that used for OA and RA have limited benefits to reduce pain and/or disease progression [14–16]. Unfortunately, nothing is known about the DS that used for Rheumatic diseases in Iraq. Therefore, the current study aimed to explore the perceptions of community pharmacists about the efficacy, safety, and quality of the DS marketed for rheumatic diseases in the Iraqi pharmaceutical market.

## Methods

### Study design

The present qualitative study was performed through face-to-face individual-based interview with community pharmacists to understand their perceptions and experience about efficacy, safety, and quality of the DS that used in treatment of rheumatic diseases (DSTRD).

The interview was accomplished through semi-structured open ended questions. Probes were applied to elicit additional comments when necessary. The interview guide (appendix A) was developed by study authors after reviewing relevant literature [17–19]. The interview guide was validated by three academic pharmacists with robust experience in qualitative research; two of them were specialized in pharmacy practice and the last one was specialized in clinical pharmacy issues.

The current study was ethically approved by the ethical committee at College of Pharmacy/University of Baghdad with an approval number of RECAUBCP712023Q at 7^th^ Jan. 2023. The ethical committee accepted to waive the written informed consent due to cultural issues and potential fear from signing documents by most Iraqi individuals and thus requested authors to obtain verbal informed consent from study participants.

### Setting and participant recruitment method

The study sample included pharmacists (with at least Bachelor degree in pharmaceutical sciences) working in community pharmacies in Baghdad, Iraq. To enroll participants a combination of convenient and purposive strategies was used [20]. The recruitment strategy was based on the participant’s gender and pharmacy location. This strategy enables the researchers to recognize the perceptions of pharmacists about DSTRD from different perspectives. To ensure sufficient experience in dealing with DSTRD and customer who use DSTRD, pharmacists with at least 6 months of working experience in a community pharmacy were considered eligible to participate in this study.

Participants were contacted before the study to inform them about the study objectives. Only those who gave their verbal informed consent (Audio-recorded) and have sufficient time for the interview were enrolled in this study. All participants were interviewed in a quiet area in their pharmacies.

### Data collection

The interviews were conducted during February 2023. The interviews were audio-recorded using iPhone 11. An approximately 10–20 minutes was needed to complete each interview. All interviews were done by the first study author after being trained on qualitative interviewing techniques through pilot interviews with community pharmacists.

Interviews were conducted till reaching the point of saturation (the point at which no new comments will be obtained). A verbatim transcription of interviews were done by the first study author and verified by the second author. All interviews were coded manually and used for the qualitative data sorting and analysis. The coding procedure was established by the second author and started by a thorough reading of each interview transcript. A codebook was developed to ensure consistent coding across interviews.

### Thematic analysis

The study researchers generated themes and subthemes using a hybrid framework (inductive and deductive) model [21, 22]. In the inductive approach, the researchers used an already prepared template based on literature for analysis of interviews. In the deductive approach, Braun and Clarke’s six steps for thematic analysis were used [23]. These steps include getting to know the comments, generating codes, searching for themes, assessing themes, defining and labelling themes, and finally writing the results. The thematic framework was validated by a panel of three experts in qualitative studies (Pharmacists with PhD degree in clinical pharmacy who published several qualitative studies).

## Results

To reach the saturation point, in-depth interviews were conducted with 20 pharmacists. Eleven of the participants were males and 9 were females. Participants’ age ranged from 23 to 60 years with a mean of 31.6 years. Regarding the academic degree of the participants, fifteen have bachelor degree, one have high diploma, two have master degree, and the last two have PhD degree in pharmacy. Participants’ working experience ranged from 0.5 to 30 years with a mean of 9.21 years. Twelve of the participants were working in pharmacies located in the south region of Baghdad, five in the west of Baghdad, two in the East of Baghdad, and the last one was working in a pharmacy at the North of Baghdad. The themes generated from this study are listed in Table 1.

**Table 1:** Study themes.

Theme	Subtheme
Pharmacist’s perception on DS	Effectiveness Safety Quality of current products Financial Impact on pharmacy business
Prescribing pattern of DS by physicians	Most commonly prescribed products Prescribing reasons
Pharmacist’s role in providing pharmaceutical care to patients prescribed DSTRD	Action of the pharmacist regarding customers requesting DSTRD Patient education on prescribed DSTRD Recommendations to improve pharmaceutical care to patients using DSTRD

### Pharmacist’s perception about the effectiveness of DSTRD

A minority of participating pharmacists (n = 6) considered DSTRD to be highly effective in achieving their intended health benefits. On the other hand, the majority of interviewed pharmacists (n = 14) reported negative concerns about the effectiveness of DSTRD. In this regard, six pharmacists reported that they consider all DSTRD to be ineffective; five pharmacists considered these supplements to be somewhat effective, and the last three pharmacists considered some products (Calcium, Vitamin D, collagen, and hyaluronic acid) to be effective while others (e.g., glucosamine and chondroitin sulfate) are ineffective. The last perception suggests that pharmacists may have different standards for evaluating the effectiveness of different supplements.

"They are not effective at all. I consider them as placebo. In practice, I did not find any patient who gets a benefit by these supplements." (Male 36 years with BSc degree and 13 years of experience)

"I found that supplements containing collagen and hyaluronic acid to be highly effective. However, I did not find any patient who gets a benefit from supplements that contain glucosamine and chondroitin." (Male 33 years with Master degree and 10 years of experience)

"They are highly effective especially products containing glucosamine and chondroitin." (Male 27 years with BSc degree and 4 years of experience)

"I think that Calcium, Vitamin D, and collagen are only 50% effective because upon aging the level of these materials is reduced in the body and thus supplementation by these products cannot solely improve the patient case." (Male 33 years with BSc degree and 10 years of experience)

### Pharmacist’s perception about the safety of DSTRD

The majority (n = 15) of the participated pharmacists perceived that DSTRD are safe and well tolerated. On the other hand, a minority of the interviewed pharmacists (n = 5) raised concerns about the safety of these products. Meanwhile, those pharmacists reported that side effects such as elevation of blood pressure (n = 3), GIT upset (n = 1), and allergy (n = 1) can be possibly occur with the usage of DSTRD.

"I consider supplements to be safe. Besides that, no any patient returns to my pharmacy complaining of side effects due to the usage of these supplements." (Female 23 years with BSc degree and 0.5 years of experience)

"Although supplements are safe, at high doses they may cause elevation of blood pressure, nausea, and vomiting." (Male 27 years with BSc degree and 4 years of experience)

### Pharmacist’s perception about the quality of DSTRD

Regarding the quality of DSTRD in the Iraqi market, six of the participated pharmacists perceived that these supplements have poor quality and five of participants considered these supplements to have good quality. On the other hand, nine of the interviewed pharmacists perceived that certain factors could influence the quality of DSTRD. For instance, five of those pharmacists believed that products manufactured by brand companies were of good quality, suggesting that reputation and brand recognition played a role in their perceptions. The last four participating pharmacists reported that DSTRD to be of reasonable quality only if they were registered and their quality assured by the Iraqi Ministry of Health (MOH), indicating that official certification and regulatory oversight were important factors in their assessment of product quality.

"The quality of DSTRD in Iraq is better than that found in neighboring countries like Turkey and Syria because most products in the Iraqi market are manufactured by brand companies. " (Male 37 years with MSc degree and 18 years of experience)

"All products are of low quality because they are not subjected to quality control assessment." (Male 33 years with BSc degree and 10 years of experience)

"The quality depends on the manufacturing company of the DS. So, products are of high quality if they are manufactured by authentic companies." (Female 26 years with BSc degree and 3 years of experience).

### Pharmacist’s perception about the financial impact of DSTRD

Most (n = 15) of the participating pharmacists perceived that selling such products have a high financial impact, indicating that they believe these products significantly contribute to their pharmacy’s revenue. On the other hand, only few participants (n = 5) mentioned that the financial impact by the selling of DSTRD is moderate.

"Any pharmacy can gain a lot of money from selling these products." (Male 40 years with PhD. Degree and 19 years of experience).

"The financial impact from selling dietary supplements is somewhat reasonable (i.e. not so high and not so low)." (Female 26 years with BSc degree and 3 years of experience).

### Prescribing pattern of DSTRD by physicians

All interviewed pharmacists reported that nearly all rheumatologists prescribe DSTRD for their patients, indicating their strong agreement on the fact that prescribing DSTRD became a common practice nowadays.

Fourteen pharmacists mentioned that products containing glucosamine were commonly prescribed, whereas 8 pharmacists believed that products containing Calcium and vitamin D were the most prescribed products. On the other hand, 6 pharmacists mentioned that the most prescribed DSTRD is Vitamin D. Other pharmacists mentioned that products containing hyaluronic acid (n = 2) and products containing Vitamin B complex (n = 2) were most seen in physician prescriptions for patients with rheumatic diseases.

"All physicians write these supplements." (Male 30 years with BSc degree and 9 years of experience)

"There is no any prescription from rheumatologists devoid from dietary supplements." (Female 25 years with BSc degree and 3 years of experience)

"Although there are many products, but physicians most commonly prescribe Osteocare and Jointace" (Male 36 years with BSc degree and 13 years of experience)

"Vitamin D is the most commonly prescribed by physician followed by Jointace." (Female 24 years with BSc degree and 1 year of experience).

Thirteen pharmacists agreed that the deal with pharmaceutical companies through medical representatives is the main reason for prescribing DSTRD. According to participants such supplements were commonly prescribed to female patients (n = 7); obese patients (n = 3); elderly patients (n = 2); pregnant woman (n = 1); patients with high educational level (n = 1); and patients with high income (n = 1).

"Prescribing dietary supplements is a common in the prescriptions of many physicians. These supplements are prescribed more commonly to females may be because of hormonal changes in women so they subjected to deficiency of certain supplements. The prescribing rate of the physician depends on the educational level of patient, so physicians prescribe supplements to those with high educational level. Meanwhile, if the physician noticed that the patient is poor then he/she will not prescribe many supplements to the patient. Anyhow, I think the deal with pharmaceutical companies is one of the main promoters for physician prescribing to these supplements. " (Male 33 years with BSc degree and 10 years of experience)

### Pharmacist’s role in caring of patients requesting DSTRD

Eight of the participated pharmacists mentioned that they directly dispense the DSTRD that requested by pharmacy customer without assessment of the patient case; however, whereas 3 of them mentioned that they dispense the requested product only if they were convinced with its efficacy. On the other hand, only five (25%) of the interviewed pharmacists mentioned that they assess the patient case by taking patient history before dispensing any supplement; this action can be seen as crucial in ensuring that the patient receives the appropriate treatment. Four (20%) of study participants reported that they ask the patient to do some laboratory tests to confirm the case before dispensing the supplement. The last three pharmacists mentioned that they refer any patient requesting DSTRD to the rheumatologist directly.

"Assess the case by taking patient history and then, and only if the case is mild I dispense the dietary supplement otherwise I refer the patient to the physician." (Female 26 years old with BSc degree in pharmacy and 3 years of experience)

"I dispense Vitamin D and glucosamine if requested by the pharmacy customer but I refuse to dispense other supplements because I do not trust in their efficacy." (Female 30 years old with BSc degree in pharmacy and 8 years of experience)

"I do not dispense the requested dietary supplement by the patient because rheumatological pain has many reasons; therefore, I usually send these patients to the laboratory to do tests for Calcium, Vitamin D, and even general urine exam. If I detect any deficiency in the laboratory data, I will dispense dietary supplements to correct this deficiency. If all laboratory data are normal, then I dispense simple dietary supplements such as Bio-Flex." (Female 24 years old with BSc degree in pharmacy and 1 year of experience)

"I usually dispense a dietary supplement if requested by any customer." (Male 43 years old with PhD degree in pharmacy and 20 years of experience)

"I do not dispense any treatment for patients with Rheumatological problems, instead I directly refer them to the physician." (Male 26 years old with BSc degree in pharmacy and 3 years of experience)

### Patient education on the dispensed DSTRD

Most of the interviewed (n = 15) pharmacists reported that they usually provide their customer with information about the prescribed supplements. Meanwhile, the duration of treatment (n = 10), method of DS administration (n = 8), dosing frequency (n = 4), possible side effects (n = 2), and possible interactions (n = 2), were the main reported information that pharmacists provide to their customers. On the other hand, 4 pharmacists mentioned that they just dispense the supplement without any further details to their customer and one pharmacist mentioned that he educates the patient by reading the physician instruction that written on the prescription only.

"I do not try to provide any information to my pharmacy customer since they just want me to dispense the prescription." (Female 28 years with high diploma and 14 years of experience)

"I will tell my patient how to use the supplement. If the supplement contains calcium, I educate the patient to avoid using iron with it. For patients prescribed Vitamin D, I educate them to take the vitamin after a fatty meal." (Male 36 years with BSc degree and 13 years of experience)

"I usually mention the duration of using glucosamine because the patient must know that he/she will not expect to get benefit from this drug by using one sheet of it. I will focus to educate the patient to continue using it for 3 months. " (Male 37 years with MSc degree and 18 years of experience)

Regarding the barriers to provide patients with information about the prescribed DSTRD, six of the interviewed pharmacists reported that there is no any barrier. On the other hand, the main barriers to communicate with patients about DS according to the interviewed pharmacists include customers feel they have sufficient knowledge about the dietary supplements (n = 10), limited trust in pharmacist competence (n = 3), low educational level of the patient (n = 1), promotion of the product in social media (n = 1), the cost of the product (n = 1), and the busy schedule of the patient (n = 1). These findings highlight the complexities of educating patients about DSTRD, and suggest that pharmacists must consider a range of factors when attempting to provide patients with information about these products.

"Some patients do not want the pharmacist to tell them anything about the dietary supplement because they have their own thoughts and ideas on this supplement." (Female 26 years with BSc degree and 3 years of experience)

"There are a lot of barriers, like the educational level of the patient. Patients with low educational level, whatever you educate them, they did not understand and instead they feel confused and leave the pharmacy while they are unsatisfied. Another barrier for patient education is the promotional videos about dietary supplements on Tiktok and facebook." (Female 30 years with BSc degree and 8 years of experience)

"The barrier between the patient and the pharmacist is so strong because of the blind trust of our patients in physician knowledge. Most patient did not believe in pharmacists competence and thus in their role to educate patients about their treatment. " (Male 26 years with BSc degree and 3 years of experience)

### Recommendations to improve pharmaceutical care for patients using DSTRD

To improve the role of pharmacists in providing optimum pharmaceutical care to customers using DSTRD only 11 (55%) of the interviewed pharmacists mentioned some recommendations such as enhancing pharmacists’ knowledge about DSTRD through medical conferences and continuous medical education programs (n = 4), pharmacists must increase the awareness of the patients about the advantages and disadvantages of DS in general (n = 3), pharmacists must follow-up patients using DSTRD (n = 1), encouraging pharmacists to cooperate with physician (n = 1), and studying the real effect of different DSTRD on Iraqi patients to provide a guide for physicians with the most effective ones (n = 2).

"Pharmacists must increase the awareness about dietary supplements on social media by providing correct information on the benefits of these supplements and the method of their use in an appropriate way." (Female 38 years with high diploma degree and 14 years of experience)

"Pharmacists must be engaged in continuous medical education programs to enhance their knowledge and hence improve their role in patient education." (Male 40 years with PhD degree and 19 years of experience)

"Really, I do not have any suggestion." (Male 30 years with BSc degree and 9 years of experience)

## Discussion

The results of this study showed that nearly half of the participated pharmacists considered DSTRD to be either ineffective or having limited effectiveness. Similarly, 52% of pharmacists in California, USA, did not trust in the effectiveness of DS [24]. On the other hand, about 1/3 of participated pharmacists in this study believed in the effectiveness of DSTRD. This finding was consistent with a survey of pharmacists working in the state of Arizona, USA, in which 32% of them reported that DS are effective [25]. Despite the similarity in beliefs of current study participants with those in USA, these beliefs contradicts the scientific literature that did not find any evidence to support the efficacy of such products [26]. The current misbelieve by 1/3 of participating pharmacists may indicate that their knowledge about DSTRD is either limited [27] or triggered by the effect of medical promotion on these products [28].

Regarding the quality of DSTRD, the results of this study showed that the majority of participated pharmacists did not believe in the quality of at least most DSTRD in the Iraqi pharmaceutical market especially if the product is not manufactured by a brand company and not registered by the Iraqi MOH. This finding is based on the fact that a large number of substandard and falsified medications are available in the private sector in Iraq [29]. In some other studies, it was found that DS do not contain the listed ingredients in the stated amounts and in some cases may contain harmful substances [30]. Moreover, the poor quality of DS products is common even in developed countries such as USA [31]. This may be because insufficient attention has been paid to supplement efficacy and there are challenges to regulatory enforcement of DS in these countries [31].

Regarding the financial impact of DSTRD, the results of this study showed that most of the interviewed pharmacists reported a high impact of selling DSTRD on their pharmacies’ income. This perception is consistent with the global increase in nutraceuticals and supplements market; such market worth almost $353 billion USD in 2019 [32]. In addition, the high prevalence of rheumatological disorder among Iraqis [33, 34] may further enhance the sell of such products.

According to the results of the present study, all interviewed pharmacists agreed on the fact that prescribing DSTRD is a common practice nowadays. This finding highlights the acceptance of these supplements by physicians especially for products containing glucosamine. This high prescribing rate for glucosamine could be attributed to the availability of some research articles that support the physician to prescribe these products by considering glucosamine to have some benefit for pain management [35]. However, meta-analysis and systematic reviews found that glucosamine has just a limited effectiveness for treatment of osteo-arthritic patients [35–37] and thus, it may be other reasons behind the high prescribing rate of DSTRD among Iraqi physicians. In this regard, the results of this study showed that most pharmacists perceived that the pharmaceutical promotion and deals on such products by pharmaceutical companies may be the main trigger for prescribing glucosamine and other DSTRD. Meanwhile, deals with pharmaceutical companies may affect the independent decision-making of physicians thus endanger the quality of patient care, reduce patients’ trust in physicians and the health system, and increase healthcare costs [38].

Regarding the prescribing of DSTRD, this study showed that 35% of the participated pharmacists mentioned that these supplements are commonly prescribed to female patients. This result is highly expected since rheumatological disorders are more common in females [39]. Regarding patient education about DSTRD, the results of this study showed that the most commonly mentioned educational point about the dispensed supplement was the duration of treatment and its method of administration, whereas dosing frequency, side effects, and interactions with the DS were the least mentioned. Similarly, many other studies found that Iraqi pharmacists mainly focus in their patient education on the method of product administration and neglect informing patients about possible interactions and side effects [40, 41].

According to the results of the present study, all participating pharmacists considered the main barrier for patient education is patient-related barriers such as low educational level of the patient, the lack of trust in pharmacists competence, and the busy schedule of the patients, besides the self-confidence of the patient in his/her knowledge about DSTRD. Similarly, other studies conducted in Sudan and Ethiopia considered patient-related factors as one of the possible barriers for patient education [42, 43]. Meanwhile, pharmacist-related barriers such as lacking competence, interest, and time were the major barriers for conducting patient education in the aforementioned studies. Unfortunately, pharmacists participating in this study neglect discussing this serious issue. Whatever the barrier for counseling and educating Iraqi patients, it is necessary for Iraqi pharmacists to join continuous medical education programs to improve their competence and skills in order to overcome these barriers [44].

In regarding the response of pharmacists to customers requesting DSTRD, the results of this study showed that most participating pharmacists directly dispense the DSTRD requested by pharmacy customers without any assessment of the case. Poor assessment of the patient case is also critiqued in many other studies evaluating the role of Iraqi pharmacist in managing minor ailments [40, 45, 46]. This poor performance of participating pharmacists may be attributed to their limited knowledge. A similar reason was reported in a recent systematic review for 19 qualitative studies conducted in both developed and developing countries [47]. Therefore, many of the participants in the present study reported that there is a need of pharmacists to join medical conferences and continuous medical education programs to enhance their knowledge, and thus improve their role in providing optimum pharmaceutical care services to Iraqi patients using DSTRD. This recommendation was also reported in other studies conducted in Iraq [40] and other developing countries [48].

To further improve pharmaceutical care, some of the current study pharmacists recommended greater pharmacist-physician cooperation mainly through studying the real effect of different DSTRD on Iraqi patients to provide a guide for physicians with the most effective ones. This recommendation is necessary to overcome the poor quality of DS in the Iraqi pharmaceutical market. In addition, participating pharmacists thought that pharmaceutical care can be improved by expanding the role of the pharmacist through conducting awareness programs for general population about the advantageous and disadvantageous of DS. Conducting such programs by pharmacists is necessary to ensure rational usage of DS by pharmacy customers [49].

The results of the present study are mainly limited by its small sample size that recruited from one governorate due to its qualitative design. Despite this limitation, the current study is the first one to be done in Iraq and its findings highlights the need for further research to identify the efficacy and quality of dietary supplements in the Iraqi pharmaceutical market.

In conclusion, most of the participating pharmacists have doubts about the efficacy, safety, and quality of DSTRD available in the Iraqi pharmaceutical market. The prescribing of such products by physicians is very common and usually triggered by deals with pharmaceutical companies. Educating patients about these supplements is very poor by most of the participating pharmacists.

## Supporting information

S1 Appendix Exploring the perceptions and dispensing practices of dietary supplements that used for rheumatic disorders in the Iraqi pharmaceutical market. (DOCX)
