# Supplementary material for: Pharmacists’ perception about efficacy, safety, and quality of dietary supplements that used for rheumatic disorders in the Iraqi pharmaceutical market
Source: PLoS One. 2024 Jul 25;19(7):e0306380. doi: 10.1371/journal.pone.0306380 (PMC11271853; doi:10.1371/journal.pone.0306380)
Supplement: S1 Appendix — (DOCX) [file pone.0306380.s001.docx]

**Appendix 1: Exploring the perceptions and dispensing practices of dietary supplements that used for rheumatic disorders in the Iraqi pharmaceutical market**

Part 1 Demographic data of participants

Age:

Gender:

College that you graduated from:

Academic degree:

Location of pharmacy:

Working experience:

Part 2: Semi structured interview guideline

1. What is your perception about the efficacy of dietary supplements for the treatment of Rheumatic disorders (DSTRD)? Can you explain?
2. What is your perception about the safety of DSFRD? Can you explain?
3. What do you think about the current prescribing rate of DSTRD by physicians? To which patients these supplements are mainly prescribed?
4. What is the main driver for prescribing of such products by physicians?
5. What are the most commonly DSTRD that recorded in physicians prescriptions? Why?
6. As a pharmacist do you think you have to provide customers with certain information about the DSTRD that you dispense? Probes: can you give me an example for the education that you provide to your customers?
7. What are the barriers to educate patients about DSTRD?
8. What is your action regarding a customer who asks you about a specific DSTRD? (Probes: Do you dispense directly, or refer to the physician, or assess the customer case before dispensing DSTRD)
9. What is the impact of selling DSTRD on pharmacy business?
10. What is your opinion about the quality of the available DSTRD in the Iraqi market?
11. What is your recommendation to improve the pharmaceutical care to customers using these supplements?
12. Any further comments?
